# Nucleobase-Containing Polymers: Structure, Synthesis, and Applications

**DOI:** 10.3390/polym9120666

**Published:** 2017-12-01

**Authors:** Haitao Yang, Weixian Xi

**Affiliations:** 1 School of Materials Science and Engineering, Nanchang Hangkong University, Nanchang 330063, China; yht@nchu.edu.cn; 2 Department of Chemical and Biomolecular Engineering, University of California Los Angeles, Los Angeles, CA 90095, USA; 3 Department of Orthopedic Surgery, University of California Los Angeles, Los Angeles, CA 90095, USA

**Keywords:** nucleobase interactions, polymer synthesis, self-assembly

## Abstract

Nucleobase interactions play a fundamental role in biological functions, including transcription and translation. Natural nucleic acids like DNA are also widely implemented in material realm such as DNA guided self-assembly of nanomaterials. Inspired by that, polymer chemists have contributed phenomenal endeavors to mimic both the structures and functions of natural nucleic acids in synthetic polymers. Similar sequence-dependent responses were observed and employed in the self-assembly of these nucleobase-containing polymers. Here, the structures, synthetic approaches, and applications of nucleobase-containing polymers are highlighted and a brief look is taken at the future development of these polymers.

## 1. Introduction

Nucleic acids in nature (DNA/RNA) represent the most powerful biomolecules. They serve as genetic material by hybridizing to a corresponding complementary strand to perform gene transcription and translation. Specifically, DNA carries genetic information from one generation to the next in living systems, and RNA guides the accurate assembly of amino acids into proteins by copying genetic information from DNA. Furthermore, in the materials science field, nucleic acids are also exploited for their selective hybridization capacities, unique geometric structures, ability to form nanoscale building blocks, directed self-assembly, and flexible programmability [1,2,3,4]. For example, DNA functionalized particles assemble into unique structures simply through the hybridization of functionalized DNA strands. This simple interaction also enables DNA nanostructures to fold into controlled shapes [5,6], nanoactuators [7,8], nanosensors [9,10,11,12], nanotweezers [13,14], and other nano-devices [15,16,17,18]. Additionally, DNA has been used as a therapeutic measure for various other biological purposes [15,16]. Despite the fact that DNA has so far enabled a handful of revolutionary advances for proof-of-concept nanomaterials, its broader impact has been limited due to restrictions imposed by the cost, scale, and scope of DNA. Thus, various attempts have been made by polymer chemists to move towards a DNA-mimicking nucleobase containing polymers [17,18,19]. In this review, we will highlight the recent advances in synthetic nucleobase-containing polymers, from their synthetic methods to their representative applications in materials science.

## 2. Nucleobase-Containing Polymers in Nature

**Figure 1:**
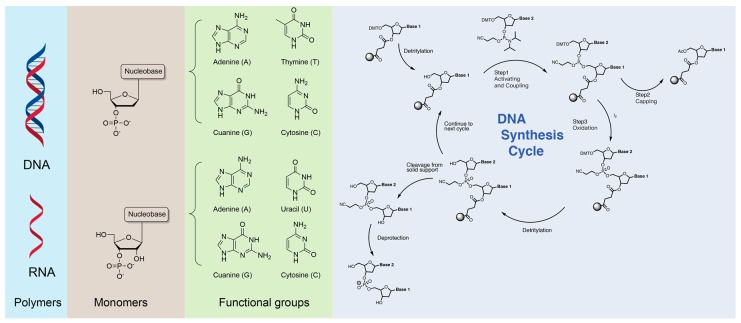
Generic chemical structures of nucleobase-containing polymers in nature and the phosphoramidite DNA synthesis cycle.

DNA and RNA are the nucleobase-containing polymers in nature. The central molecular interaction between these polymers is a hydrogen bond between corresponding nucleobases; these nitrogenous bases are adenine (A), guanine (G), cytosine (C), thymine (T), and uracil (U). DNA usually forms duplexes that are stabilized by hydrogen bonding between adenine (A) and thymine (T); cytosine (C) and guanine (G). RNA, in most cases, consists one strand of nucleic acids, using uracil (U) instead of thymine (T) in its nucleobase pairing [20]. Besides the biosynthesis of DNA/RNA in nature, oligonucleotide synthesis is based on phosphoramidite chemistry, which was developed in the 1980s and later combined with solid-phase supports and automation [21,22] (Figure 1). However, the length of synthetic oligonucleotide is limited by the coupling efficiency and the following purification steps, which resulted in a typical length of synthetic single-stranded DNA/RNA of around 70~100 bases, with low error rates [23]. Most recently, Zauscher et al. utilized transferase-catalyzed living chain-growth polycondensation to synthesize well-defined natural DNA sequences such as homopolynucleotides (poly(dT), poly(dA)), random polynucleotides, and block polynucleotides with more than 1000 bases. This work is an excellent example of employing enzymatic living polymerization to achieve DNA synthesis in the absence of a DNA template [24]. 

## 3. Synthetic Nucleobase-Containing Polymers

To mimic DNA/RNA sequences in nature and implement their sequence recognition behaviors in polymers chemistry and materials science, synthetic chemists have chosen nucleobases as a “smart” functional group in the side chain of their polymers. Furthermore, with the development of various polymer synthesis skill sets, especially controlled living polymerization, more and more efforts have been made toward nucleobase-containing polymers synthesis and many of them have been exploited into self-assembly polymeric structures such as micelles or nanogels based on specific nucleobase interactions. In subsequent sections, we will highlight different nucleobase-containing polymers based on different polymerization mechanisms and discuss representative examples of each kind of nucleobase-containing polymer in various polymer science applications. The common methods for preparation of NB-containing polymers were showed in Table 1.

### 3.1. Synthesizing Nucleobase Polymers by ATRP

**Scheme 1:**
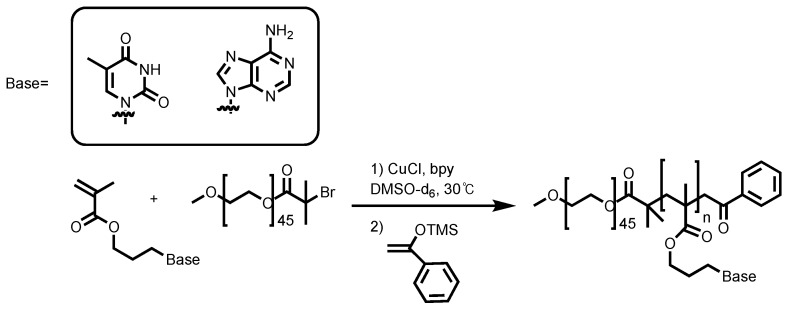

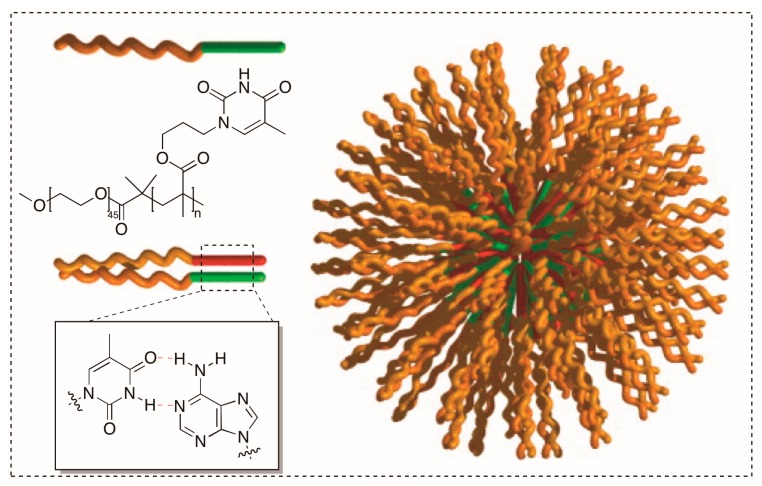
Synthesis of copolymer poly(ethylene glycol) (PEG)–poly(nucleobase) by ATRP and schematic structure of assembled copolymers [26]. Reproduced with permission. Copyright John Wiley and Sons, 2006.

Since Atom Transfer Radical Polymerization (ATRP) was independently discovered by Sawamoto and Matyjaszewski et al. [45,46] in 1995, by virtue of affording polymers of narrow molecular weight distributions, high molecular weight, and advanced macromolecular architectures, it presents a powerful method to prepare a great variety of polymers, including nucleobase-contained polymers. Spijker et al. utilized ATRP to synthesize a series of well-defined poly(ethylene glycol), (PEG)–poly(adenine) and PEG–poly(thymine) nucleobase containing block co-polymers from a PEG-based macroinitiator [26]. Nucleobase-functionalized monomer (thymine and/or adenine), catalyst (CuCl), ligand (bpy) and PEG-based macroinitiator (stoichiometric) were mixed and underwent typical ATRP procedures. The expected conversion was analyzed by ^1^H-NMR spectroscopy. Finally, the reaction was quenched by 1-phenyl-1-(trime-thylsiloxy)ethylene (showed in Scheme 1). Furthermore, to demonstrate the unique properties of the nucleobases in the sidechain, they investigated the aggregation behavior of two complementary block copolymers in water (The structure of assembled copolymers is shown in Scheme 1). They found an interaction between the complementary thymine and adenine moieties, which resulted in an increased solubility of the block copolymers by an improved shielding of the hydrophobic blocks.

In 2007, the same group reported the synthesis and controlled polymerization of all four nucleobases’ (thymine, adenine, cytosine, and guanine) methacrylate monomers through ATRP method [25]. Technically, by alkylation of the nucleobases with 3-bromopropyl methacrylate, thymine-, adenine-, cytosine-, and guanine-functionalized methacrylate monomers were prepared and their ATRP kinetics were examined. Interestingly, they found the formation of guanine and cytosine monomer with copper(II), and these two specific polymerizations required more addition of catalyst and the use of PMDETA. From this study, all four unprotected nucleobase monomers could be polymerized in a controlled manner, but there is still space for improving the level of control in the synthesis of nucleobase-containing polymers through ATRP. 

**Scheme 2:**
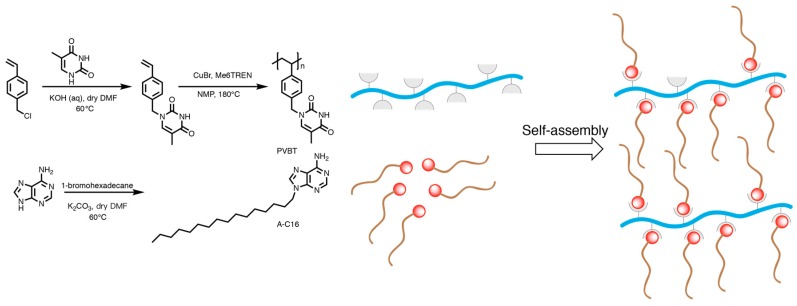
Synthetic procedures used to obtain PVBT and A-C16 and graphical representations of the lamellar structures of the PVBT/A-C16 complexes in bulk state [47]. Reproduced with permission. Copyright John Wiley and Sons, 2008.

Similarly, Cheng et al. [47] synthesized poly[1-(4-vinylbenzyl)thymine] (PVBT) through ATRP at a high temperature (showed in Scheme 2) and, furthermore, performed nucleobase recognition through adding presynthesized 9-hexadecyladenine (A-C16) in the presence of polythymine homopolymers. Specifically, due to the hydrogen bonding interactions between the thymine groups of PVBT and the adenine group of A-C16, the biocomplementary PVBT/A-C16 hierarchical supramolecular complexes were formed in dilute DMSO solution. In the bulk state, these complexes self-assemble into well-ordered lamellar structures; these self-assembled structures can be tuned by different A-C16 loadings. In 2015, a water-soluble triblock polymer and a pentablock-polymer containing nucleobases were synthesized by Haddleton and Garcia et al. [27]. They utilized ATRP to polymerize methacryloyl uridine and methacryloyl adenosine from bromoisobutyroyl poly(ethylene glycol) (PEG) macroinitiators. Furthermore, they investigated the secondary structures of triblock polymers and pentablock polymers in water by CD spectrum and mixing adenosine and uridine triblock copolymers in water resulted in a new supermolecular self-assembled structures, which provided direct proof of hydrogen bonding interaction between nucleobases.

### 3.2. Synthesizing Nucleobase Polymers by RAFT

As popular as ATRP, the RAFT (reversible addition-fragmentation chain transfer) process, which was first introduced by the group of Rizzardo [48] in 1998, is also recognized as one of the most versatile methods for producing a wide range of macromolecules with well-defined functionalities and complex architectures.

**Figure 2:**
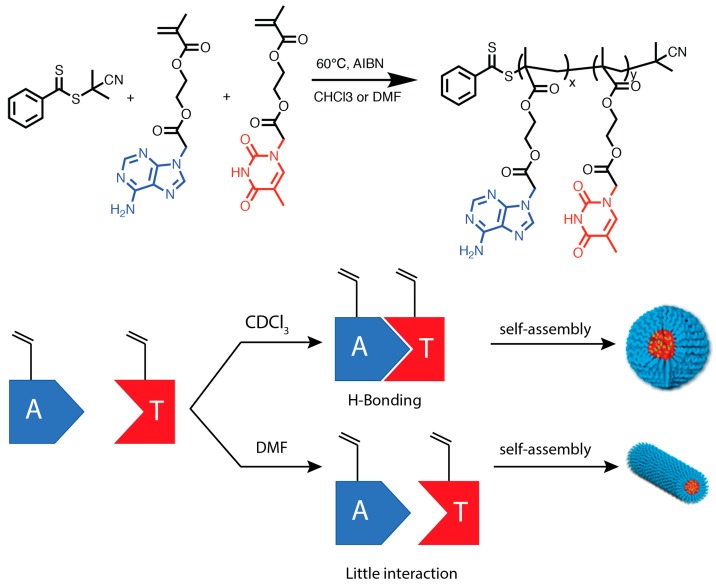
Synthesis and self-assembly of adenine and thymine block copolymers using RAFT [49]. Reproduced with permission. Copyright American Chemical Society, 2013.

In 2013, Kang et al. synthesized methacryloyl-monomers containing adenine and thymine to yield homopolymers and copolymers with excellent control over molecular weight and end group fidelity via RAFT polymerization (shown in Figure 2) [49]. Their results indicated that the polymerization solvent greatly affected the monomer reactivity; for example, chloroform promoted hydrogen bonds between the adenine monomers and thymine monomers, preferring moderate alternating copolymers, while solvent DMF suppressed these interactions, preferring statistical copolymers. Furthermore, they investigated the self-assembly properties of the copolymers and their TEM and DLS results clearly indicated that the presence or absence of hydrogen bonding between nucleobases could influence the morphology of forming micelles. Similarly, the same group explored more nucleobase-containing materials via RAFT dispersion polymerization in 1,4-dioxane and chloroform, and also investigated the behavior of copolymers self-assembly [29]. The results confirmed again that the morphologies of self-assembled nucleobase-containing polymers were dependent on polymerization solvents. For example: spherical micelles with internal structures were only observed in chloroform by varying the *DP* of the core-forming block. As an extension of this work, in 2015 they reported the synthesis and self-assembly of thymine-containing polymers via RAFT dispersion polymerization and a wide variety of morphologies were accessed by changing the different solvent of polymerization or incorporating adenine-containing mediators [50]. These results strongly demonstrated that nucleobase interactions played a central role in controlling the shape and size of polymeric self-assemblies.

**Scheme 3:**
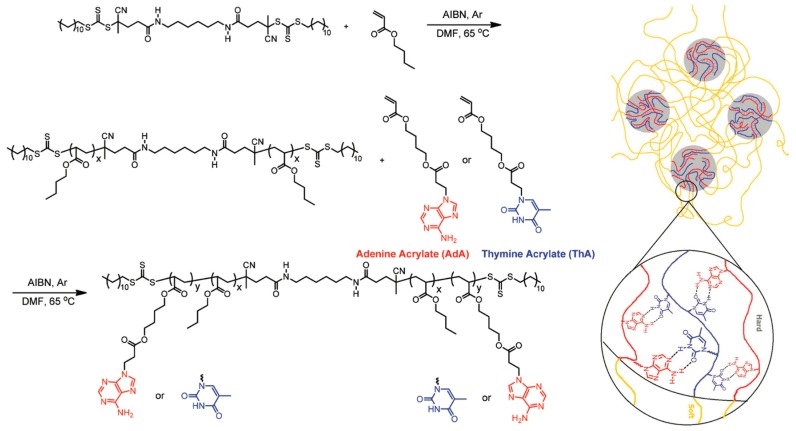
Synthesis of adenine- and thymine-functionalized acrylic ABA triblock copolymers using RAFT polymerization and schematic representation of supramolecular blend of adenine and thymine-functionalized triblock copolymers [31]. Reproduced with permission. Copyright Royal Society of Chemistry, 2015.

Zhang et al. used a similar RAFT polymerization strategy to synthesize well-defined acrylic ABA triblock copolymers with nucleobase-functionalized external blocks and poly(*n*-butyl acrylate) (PnBA) as a central block (shown in Scheme 3) [31]. Moreover, they prepared triblock copolymer films and supramolecular blend and their results indicated that the acrylic monomer with a flexible spacer to the pendant nucleobases promoted intermolecular recognition of nucleobases, lowering the *T*_g_ of functionalized polymers. In addition, the complementary hydrogen bonding between adenine and thymine formed a thermally labile, physically crosslinked network that exhibited enhanced mechanical performance with melt processability, and the supramolecular blend exhibited a cylindrical microphase-separate morphology. More and more work has been using RAFT as an efficient and robust method for the preparation of multi-block polymers with nucleobase functionalities, and more and more morphologies have been accessed by fine tuning of the interactions between nucleobases [30,32,51]. RAFT has been the most frequently used living polymerization method in preparing nucleobase-containing polymers; however, it is still worth noting some challenges of this method. For example, RAFT usually requires careful selection of chain transfer agents (CTAs) for each monomer, which may require screening of CTAs before the polymerization of different nucleobase-containing monomers.

### 3.3. Synthesizing Nucleobase Polymers by NMP

**Figure 3:**
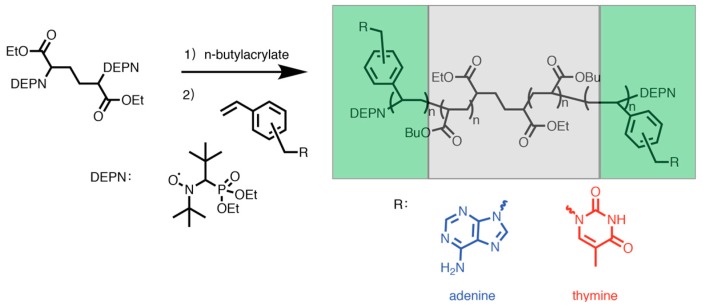
Synthesis of adenine and thymine nucleobase-functionalized triblock copolymers using NMP [33]. Reproduced with permission. Copyright American Chemical Society, 2007.

As we described before, the coordination of nucleobase (guanine and cyotsine) with a metal catalyst (e.g., CuBr) usually attenuates the polymerization kinetics of ATRP. As such, nitroxide-mediated polymerization (NMP) could be utilized as another controlled radical polymerization (CRP) technique to produce nucleobase-containing polymers [52,53]. Mather et al. utilized a novel difunctional alkoxyamine initiator to polymerize styrenic nucleobase monomers with poly(*n*-butyl acrylate) homopolymers via a two-step NMP route [33] (Figure 3). The resulting triblock copolymers contain either adenine or thymine as outer blocks, and *n*-butyl acrylate rubber as inner blocks. Once adenine and thymine polymers are mixed in chloroform, the viscosity of the blend increased dramatically, indicating the existence of hydrogen-bonding interactions between complementary nucleobase subunits since viscosity is a function of effective molecular weight. Furthermore, the annealed mixture in the solid state revealed a higher softening temperature and presented further proof of a complementary A–T hard phase. Although the NMP method avoids using a metal catalyst in polymerization, it still has some inherent challenges that limit its wide application in preparing nucleobase-containing polymers. For example, the slow polymerization kinetics usually requires high temperatures and a long polymerization time.

### 3.4. Synthesizing Nucleobase Polymers by Conventional Radical Polymerization

Besides controlled polymerizations, conventional radical polymerization also allows efficient large-scale synthesis of nucleobase functionalized polymers. As early as 2005, Luts et al. investigated the polymerization of a series of monomers bearing nucleobases 1-(4-vinylbenzyl)uracil (VBU), 1-(4-vinylbenzyl)thymine (VBT), and 9-(4-vinylbenzyl)adenine (VBA) [37]. The corresponding homopolymers (PVBT, PVBU, and PVBA) were prepared in high yields via conventional radical polymerization, while the copolymers of dodecyl methacrylate (DMA) with either VBT or VBA were prepared by ATRP and free radical polymerization. The results indicated that these homopolymers were not very practical due to poor solubility in most of the common organic solvents. However, copolymers of DMA with either VBT or VBA could be dissolved in a range of organic solvents. Furthermore, the formed copolymers P(VBT-*co*-DMA) and P(VBA-*co*-DMA) were found to self-assemble in dilute solutions in dioxane or chloroform, as confirmed by the hypochromicity effect in UV spectroscopy. Nevertheless, it was found that at higher concentrations of chloroform, P(VBT-*co*-DMA), P(VBA-*co*-DMA), or P(VBT-*co*-DMA)/P(VBA-*co*-DMA) mixtures spontaneously self-assembled into spherical aggregates. Later in 2006, the same group studied in detail the self-organization of random copolymers composed of a nucleobase monomer (either VBT or VBA) and DMA in dilute chloroform [35]. They investigated how the balance between the molar fractions of the nucleobase monomer and DMA could affect the solubility and self-assembly behavior of nucleobase-containing copolymers. In addition, they demonstrated that hydrogen bonding was an efficient driving force for organizing synthetic nucleobase-containing polymers in a noncompetitive solvent.

**Scheme 4:**
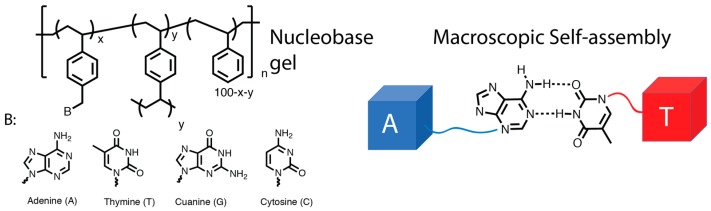
Chemical structures of the nucleobase organogels and a schematic illustration of their macroscopic self-assembly [36]. Reproduced with permission. Copyright John Wiley and Sons, 2015.

Nakahata presented an interesting work that created a selective macroscopic self-assembly system by using polymer gels modified with either complementary DNA oligonucleotides or nucleobase-containing polymers [36]. Specifically, hydrogels and organogels were obtained via conventional radical terpolymerization of AAm or styrene (main chain units) and MBAAm or DVB (crosslinking units) (showed in Scheme 4). The oligonucleotide-modified hydrogels exhibited macroscopic recognition between complementary strands. Similarly, nucleobase-modified organogels exhibited the same adhesion as DNA oligonucleotide-functionalized hydrogels and were visualized on a macroscopic scale. Direct adhesion between macroscopic objects by nucleobase interaction provides the possibility of creating “smart” materials that can bind many kinds of heterogeneous materials without adhesives. Although conventional radical polymerization provides a very simple method for nucleobase-containing polymer synthesis (no metal catalyst, no precursors), it also has disadvantages such as insufficient control of the polymer length, polydispersity, and macromolecular structure, which may impair its potential applications in biology and materials science.

**Figure 4:**
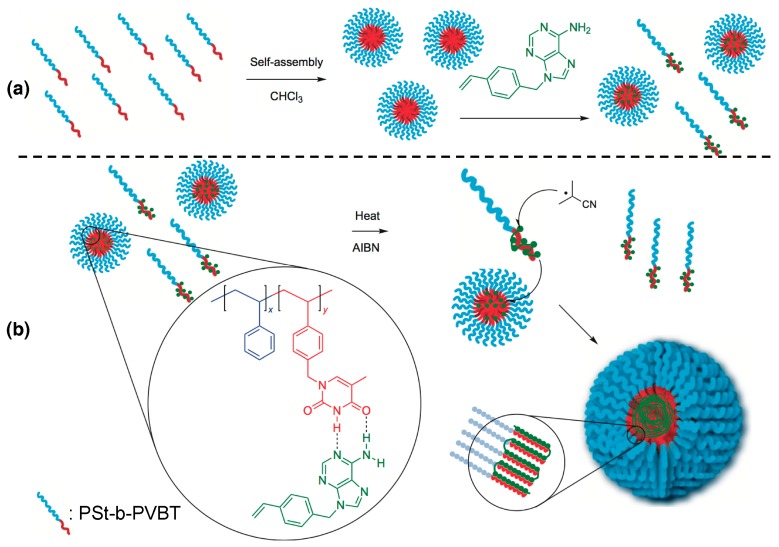
(**a**) Self-assembly of nucleobase polymer templates into micelles and (**b**) the templated polymerization of complementary nucleobase monomers [28]. Reproduced with permission. Copyright Springer Nature, 2012.

With the inherent property of sequence recognition, McHale et al. reported that the nucleobase core of a block copolymer micelle can serve as a template for controlled radical polymerization [28] (Figure 4). This uniquely templated polymerization yields unexpectedly high molecular weight polymers (up to ~400,000 g/mol), with low polydispersity (*P* ≤ 1.08); and their TEM imaging clearly demonstrated that polymerization takes place in the core of the micelle as its size changes from ~13 to ~20 nm before and after polymerization. More interestingly, the discrete micelle core structures enabled the control of the polymerization by segregation of propagating radicals.

### 3.5. Synthesizing Nucleobase Polymers by Click Chemistry

**Figure 5:**
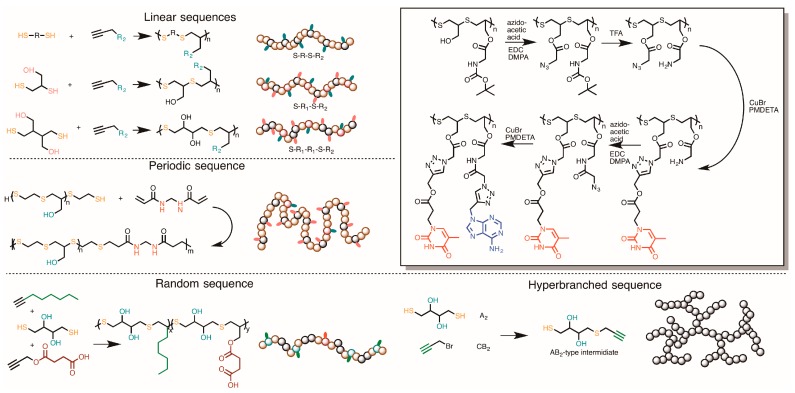
Sequence-controlled hetero-functional, topological polymers (R_1_ and R_2_ denote two different functional groups and S represents a thioether bond, which can be transformed into a sulfone bond by oxidation) and the synthetic route to a DNA-mimetic nucleobase-containing polymer [39]. Reproduced with permission. Copyright Springer Nature, 2014.

“Click chemistry” is a concept introduced by Sharpless [54] in 2001 and since then has become one of the most powerful paradigms in polymer chemistry and materials science [55,56,57]. Due to its simplicity and efficiency, click chemistry has been extensively employed for accessing linear polymers [58,59,60,61,62,63], branched polymers [64,65,66,67,68,69,70,71,72,73], dendrimers [74,75,76,77,78,79], and polymer networks [80,81,82,83,84,85]. The combination of click chemistry and nucleobase-containing polymers is promising. For example, in 2014 Han et al. reported a facile and scalable method to synthesize DNA-mimetic sequential polymers with tailored hetero-functional groups based on radical-initiated thiol-yne click polymerization [39]. Firstly, radicals were generated from either a UV or thermal initiator, yielding a thiyl radical, enabled stepwise propagation, and polymer chain transfer (shown in Figure 5). With a delicate selection of monomers with different functionalities, various types of sequenced polymers such as linear sequence, periodic sequence, random sequence, and hyperbranched sequence were obtained (Figure 5). Furthermore, to achieve single-strand DNA-mimetic macromolecules, alternating adenine and thymine groups were introduced by another classic click chemistry: Cu-catalyzed azide and alkyne cycloaddition (CuAAC). This work is an excellent example of utilizing two orthogonal click reactions to synthesize nucleobase-containing polymers from simple starting substrates in two simple steps, indicating the power of click chemistry.

**Scheme 5:**
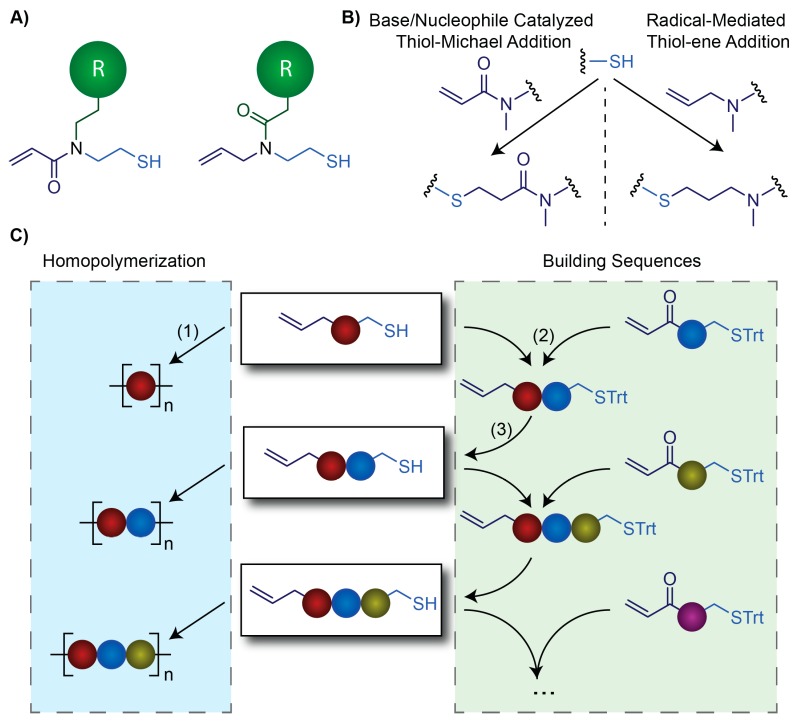
(**A**) General monomer structure for thiol-based click polymerization (**R** represents functional group, including but not limited to nucleobases); (**B**) The two types of thiol click reaction; (**C**) Thiol-click reaction strategy for sequence-controlled periodic functional oligomers and polymers [38]. Reproduced with permission. Copyright John Wiley and Sons, 2015.

In 2015, Xi et al. [38] synthesized nucleobase-containing sequence-controlled polymers (homopolymers of a single nucleobase or homopolymers of specific repeating nucleobase sequences) by combining orthogonal thiol-ene and thiol-Michael click chemistry reactions (Scheme 5) [86,87,88,89]. In this case, a thiol-protected acrylamide monomer is coupled to a functionalized thiol–allylamine monomer in a thiol-Michael addition, then the thiol group is deprotected to restore the thiol and polymerized with allyamine functional groups by radical initiation of the thiol-ene reaction. This “click” by “click” approach is a great demonstration of synthesizing nucleobase-functionalized sequence-controlled periodic copolymers/oligomers and polymers without the use of a solid support. In addition, these nucleobase-containing polymers were employed in various applications in materials science, such as sequence-controlled assembly of nanoparticles, DNA delivery [90], and organogel formation [91].

**Figure 6:**
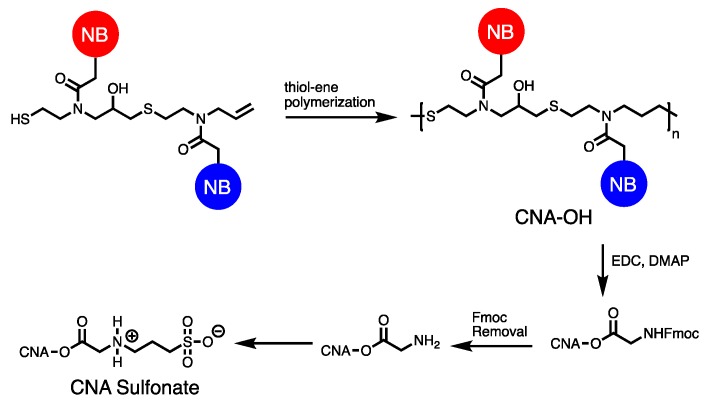
Schematic illustration of water-soluble CNA polymer synthesis [40]. Reproduced with permission. Copyright Royal Society of Chemistry, 2017.

In 2017, Liu et al. [40] presented a synthesis of an intrinsically water-soluble single-stranded DNA analog formed by the synthesis of a Clickable Nucleic Acid (CNA). They formed molecules with pendant hydroxyl groups present on the main polymer backbone, and subsequent modification of those hydroxyls with sulfonate moieties further enhances the hydrophilicity (Figure 6). The CNA polymer with one pendant hydroxyl group for each dimer was obtained by the radical-mediated thiol-ene polymerization approach. A few simple steps were needed to functionalize the free hydroxyl group on the polymer backbone to obtain the water-soluble CNA polymer. Furthermore, they demonstrated that this water-soluble CNA polymer was capable of binding to complementary DNA on the surface of nanoparticles. Although click chemistry enables fast and efficient preparation of nucleobase-containing polymers, there is still a lot of space for researchers to optimize synthetic strategies/procedures to achieve longer polymers and sequence control in the future.

### 3.6. Synthesizing Nucleobase Polymers by ROMP

**Figure 7:**
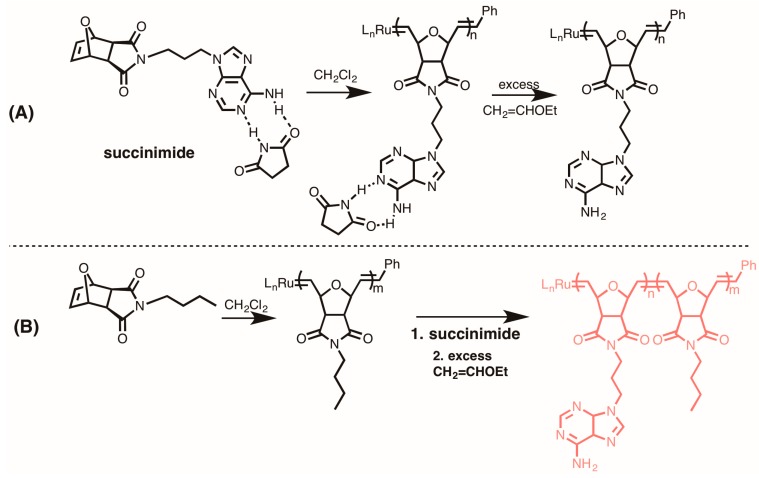
(**A**) ROMP with noncovalently protected monomer; (**B**) preparation of block copolymers via ROMP [94]. Reproduced with permission. Copyright American Chemical Society, 2006.

As a variation of the olefin metathesis reaction, ring-opening metathesis polymerization (ROMP) [92,93] represents another widely used method to synthesize polymer materials with tunable sizes, shapes, and functions, especially in the preparation of nucleobase-containing polymers. Bazzi et al. [94] first reported the preparation of “DNA-mimetic” homopolymers and block copolymers through controlled ring-opening metathesis polymerization of non-covalently protected monomers as early as 2002. Notably, an adenine-containing monomer was “protected” by succinimide, based on the hydrogen bonding between succinimide and the adenine moiety. Additionally, this simple introduction of succinimide increased the solubility of the resulting polymer, which prevents resultant polymers from precipitating before the reaction is complete. These nucleobase-containing block copolymers were produced by following the same ROMP procedure. Finally, the self-assembly of copolymers (*m* = 50, *n* = 5; shown in red in Figure 7) was investigated in THF solution and the results revealed the ability of these hydrogen-bonding-enabled self-complementary block copolymers to self-assemble into novel nanoscale rod morphologies.

**Scheme 6:**
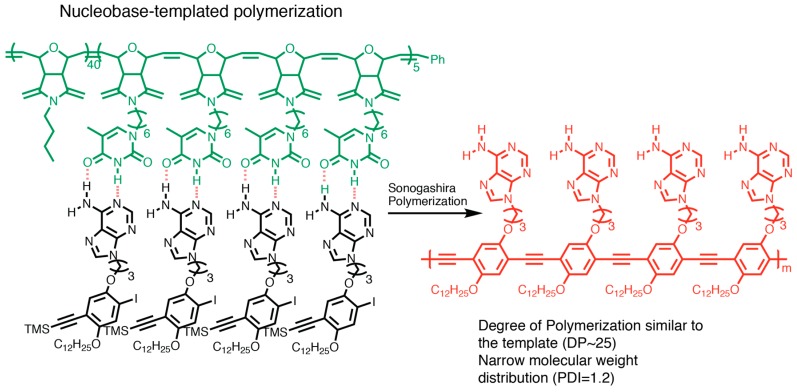
Templated polymerization of nucleobase-containing monomers [41]. Reproduced with permission. Copyright American Chemical Society, 2009.

After that, a similar ROMP strategy was utilized by Lo et al. [41] in the construction of a thymine-containing block copolymer with narrow molecular weight distribution. As shown in Scheme 6, adenine-containing monomers were aligned with hydrogen-bonding interactions to the thymine template, and subsequently underwent Sonogashira polymerization, leading to the templated synthesis of a conjugated polymer. The daughter strand is found to possess a narrow molecular weight distribution and a chain length nearly equivalent to that of the parent template, indicating that the daughter strand could copy the length and distribution information from the template strand. However, non-templated polymerization or polymerization with the incorrect template generated a short conjugated oligomer with a significantly broader molecular weight distribution.

**Figure 8:**
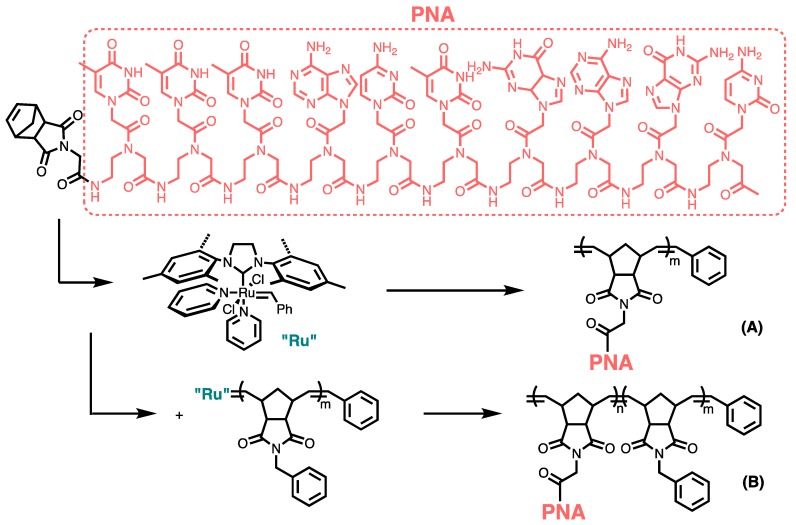
Preparation of poly(oligonucleotide): PNA homopolymer (**A**); Poly-PNA block copolymer (**B**) [42]. Reproduced with permission. Copyright American Chemical Society, 2014.

Peptide nucleic acids (PNAs) are synthetic DNA mimetics whose backbones are electrical neutral amide bonds, which contribute high stability towards nuclease degradation and high affinity towards complementary DNA/RNA strands [95,96,97,98]. James et al. [42] reported a preparation of polymeric nucleic acids wherein single-stranded sequences of PNAs are incorporated as polymer brushes via graft-through polymerization using the ROMP initiator “Ru”. This work clearly demonstrated the power of ROMP in preparing amphiphilic brush copolymers by graft-through polymerization of a relatively large side chain: oligonucleotides (Figure 8). This approach of combining PNAs and ROMP could provide an efficient strategy for the incorporation of nucleic acids into particles and polymer-based materials, which might be useful for various applications such as affinity purification of DNA, gene delivery, and programmable materials. Like other polymerization strategies, ROMP provides another efficient method to access nucleobase-containing polymers; however, the driving force of ROMP is the relief of ring strain in cyclic monomers, which usually requires the additional step of preparing nucleobase-functionalized cyclic olefins before polymerization. The cost of the Ru catalyst is another concern, especially in large-scale polymer preparations.

Most recently, Gregory et al. [99] presented a novel work that using a ring-opening polymerization method other than ROMP. In this work, thymidine-based cyclic carbonate incorporating CO_2_ at 1 atm pressure and this monomer underwent ROP under mild reaction conditions with organocatalyst TBD and 4-MeBnOH alcohol initiator to yield novel thymidine-based polycarbonates. Similarly, Tsao [43] et al. presented a synthetic strategy that employed organocatalytic ROP of a six-membered cyclic phosphoester monomer at ambient temperature to afford a new type of thymidine-derived 3′,5′-linked polyphosphoester with a butenyl group located on each repeat unit. This is the first time such well-defined DNA-derived polyphosphoesters with 3′,5′-linkages in the backbones have been prepared via ROP.

### 3.7. Synthesizing Nucleobase Polymers by Other Polymerizations

**Figure 9:**
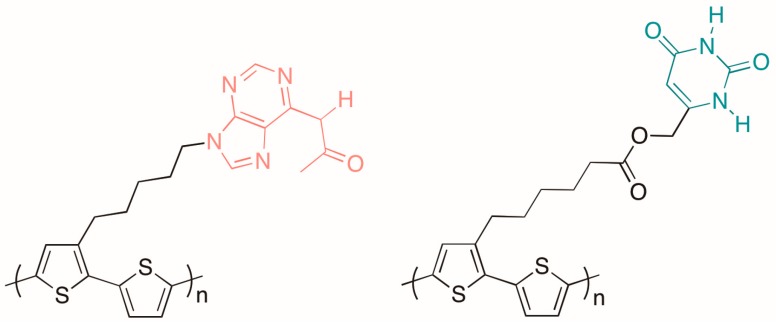
Structures of nucleobase-functionalized polythiophenes [44]. Reproduced with permission. Copyright John Wiley and Sons, 1998.

The nucleobase chemistry has also been overlapped with conducting polymers. Bäuerle et al. [44] presented the synthesis, characterization, and polymerization of bithiophenes with the nucleobases uracil and adenine as sidechains, and found the molecular recognition could influence the electronic properties of the polythiophenes, e.g., the presence of a complementary base could induce an increase in the oxidation potential for polymers (Shown in Figure 9).

## 4. Applications of Nucleobase-Containing Polymers in Materials Science

### 4.1. Hydrogels

**Scheme 7:**
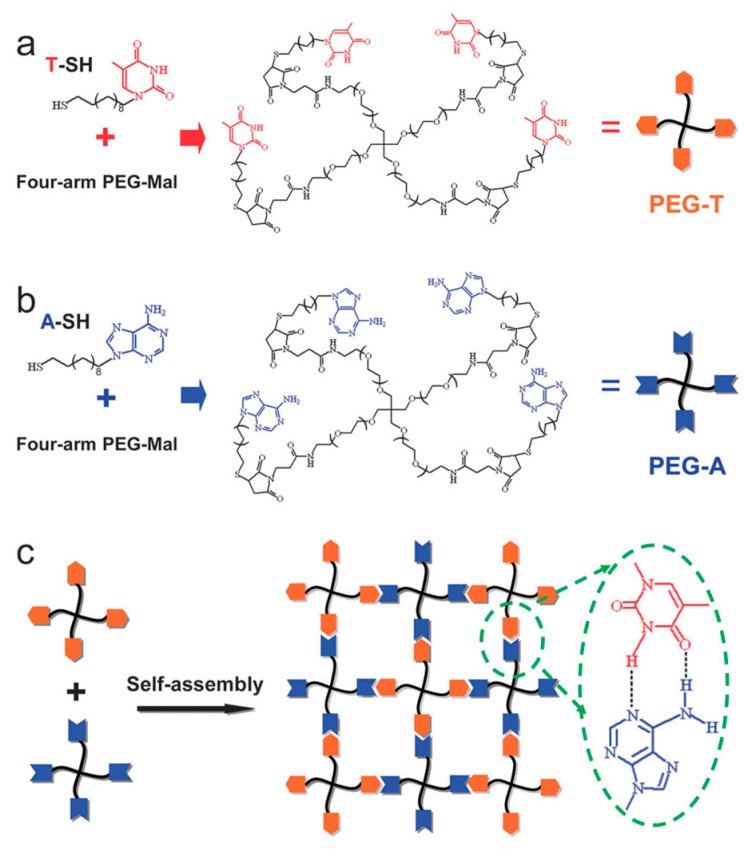
Poly(ethylene glycol)-maleimide (PEG-Mal) was functionalized with (**a**) thiol thymine (T-SH) and (**b**) thiol adenine (A-SH); (**c**) Schematic of the biological self-assembly of ideal four-arm PEG hydrogel network via intermolecular nucleobase interaction between thymine and adenine functionalities [103]. Reproduced with permission. Copyright Royal Society of Chemistry, 2012.

Hydrogels have served as matrices for 3D cell culture with widespread applications in tissue engineering, biomolecule delivery, and regenerative medicine [100,101]. Based on the crosslinking mechanism, hydrogels can be classified into physically crosslinked hydrogels and chemically crosslinked hydrogels. Although chemically crosslinked hydrogels usually provide a stable polymer network with slow degradation kinetics, physically crosslinked hydrogels have also attracted a lot of attention, especially as injectable biomaterials in translational medicine [102]. Among all the physical crosslinked mechanisms for hydrogel formation, hydrogen bonding remains one of the most frequently used methods, which makes nucleobase-containing polymers an ideal candidate for physically crosslinked hydrogels. In 2012, Tan et al. developed a biological hydrogel that self-assembled via nucleobase interaction from adenine- and thymine-functionalized tetra-arm PEG polymers (Scheme 7) [103]. This hydrogel exhibited both thermal and mechanical responsiveness, such as gel-sol transition under high strain conditions or high temperature, which could break the physical linkage between adenine and thymine. Additionally, they demonstrated that this nucleobase-interaction-based hydrogel can serve as an injectable scaffold for growth factor delivery and cell encapsulation, which indicated the excellent biocompatibility of these materials and their potential for use in further biomedical applications.

### 4.2. DNA Delivery

**Figure 10:**
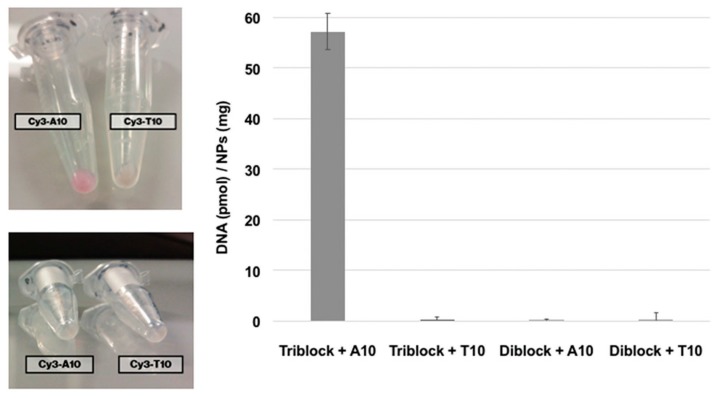
DNA loading measurement of triblock PEG-CNA-PLGA nanoparticles [90]. Reproduced with permission. Copyright John Wiley and Sons, 2017.

DNA delivery, especially non-viral DNA delivery, is a powerful and popular tool for gene silencing in various diseases [104,105,106,107]. However, there are still challenges to building an efficient delivery system. In most cases, cationic polymers have been widely studied and investigated for DNA delivery, but they usually encounter some difficulties such as low DNA uptake, slow DNA release, and lack of nuclear targeting [108,109]. To expand the materials toolbox for DNA delivery, Harguindey et al. utilized synthetic DNA analogs (CNAs) and synthesized triblock copolymer (PEG-CNA-PLGA) for sequester high loading of DNA [90]. These synthetic triblock copolymers were translated into polymer nanoparticles through an emulsion technique. In addition, these CNA-containing particles exhibited high encapsulation of complementary DNA sequences through specific nucleobase recognition interactions, which demonstrated the potential application of these CNA-containing particles as DNA carriers for specific gene silencing (Figure 10).

### 4.3. Self-Healing Materials

**Figure 11:**
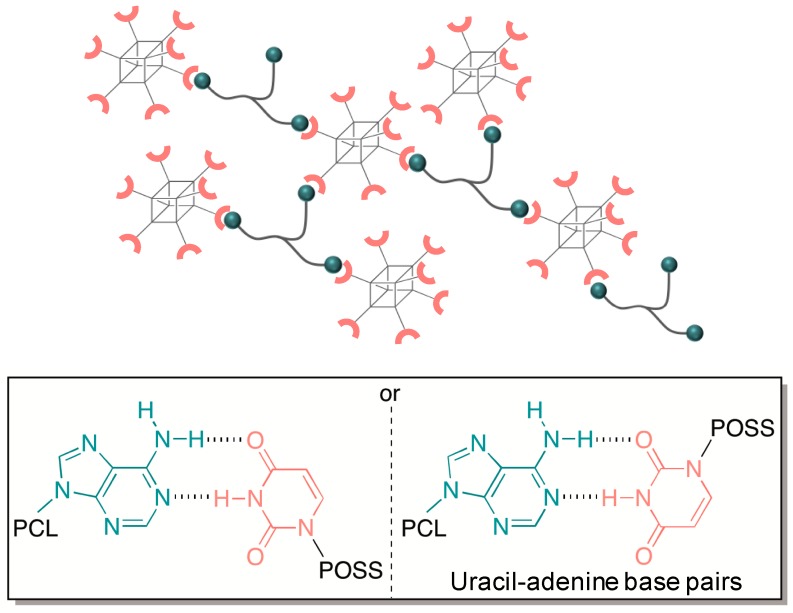
Structures of nucleobase-containing polymers and an illustration of complementary interactions between POSS-U and PCL-A [116]. Reproduced with permission. Copyright Royal Society of Chemistry, 2015.

Self-healing materials include but are not limited to metals, polymers, and composites that have the capability to heal and recover the original state after damage (e.g., mechanical, thermal) [110,111]. Over the last decade, they have attracted much attention and been investigated intensely [112,113,114,115]. Depending on the nature of the self-healing process, it can be classified into two types: autonomic and non-autonomic [110]. Specifically, non-autonomic self-healing materials need external energy, while autonomic materials do not require extra stimulation for self-healing. Complementary nucleobase-containing polymers have a built-in advantage in the preparation of materials that exhibit tunable mechanical properties and self-healing behaviors, providing an efficient potential route to the fabrication of multifunctional materials. Cheng [116] et al. developed a multi-uracil functionalized polyhedral oligomeric silsesquioxane (POSS-U) and adenine end-capped three-arm polycaprolactone oligomer (PCL-A), as showed in Figure 11. Their materials showed a good complementary ability either in solution or solid state due to the uracil (U)–adenine (A) cross-linking. In addition, the prepared film materials could self-heal autonomously at room temperature without external intervention, which demonstrated a simple and efficient way to build a series of self-healing materials for a wide range of potential applications.

**Figure 12:**
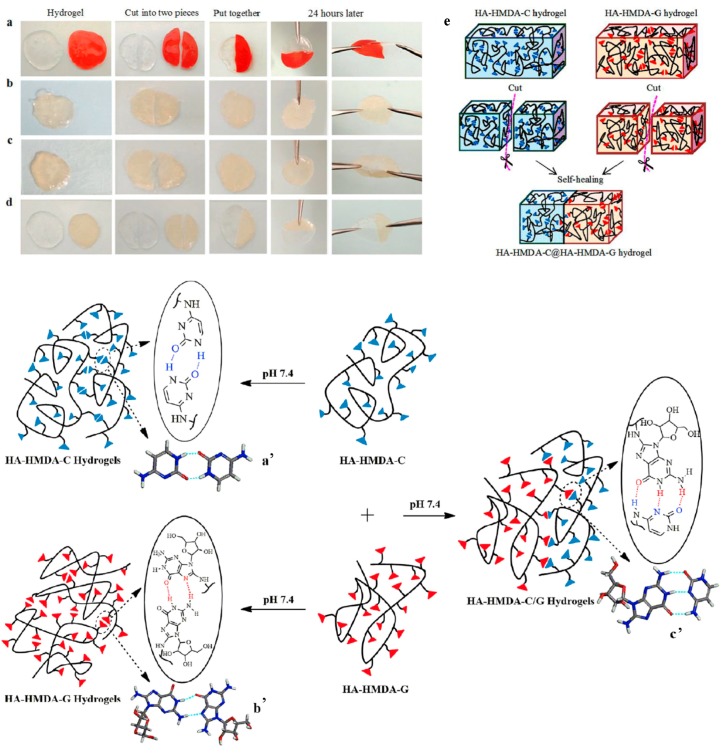
Illustration and diagram of self-healing behavior of (**a**) HA-HMDA-C10, (**b**) HA-HMDA-G10, (**c**) HA-HMDA-C/G10, (**d**) HA-HMDA-C10@HA-HMDA-G10 hydrogels, and (**e**) simulation diagram of HA-HMDA-C10@HA-HMDA-G10. The sketches are of hydrogen bonds between cytosine and guanosine, with themselves or each other, carried out by computer simulations: (**a’**) cytosines, (**b’**) guanosines, and (**c’**) cytosine and guanosine [117]. Reproduced with permission. Copyright Elsevier, 2017.

Ye et al. [117] utilized nucleobase interactions as another example of preparing self-healing pH-sensitive biodegradable cytosine and guanosine-modified hyaluronic acid hydrogels (HA-HMDA-C, HA-HMDA-G, and HA-HMDA-C/G) under physiological conditions (Figure 12). Their results indicated that all materials have good self-healing ability, but materials with a high concentration of HA had a short gelation time and strong mechanical properties. Furthermore, the mechanical properties of HA-HMDA-C/G hydrogels were better than those of C–C or G–G, which confirmed that the nucleobase interactions between C and G are stronger than the other two types in this case.

### 4.4. Self-Assembly of Nanoparticles

**Figure 13:**
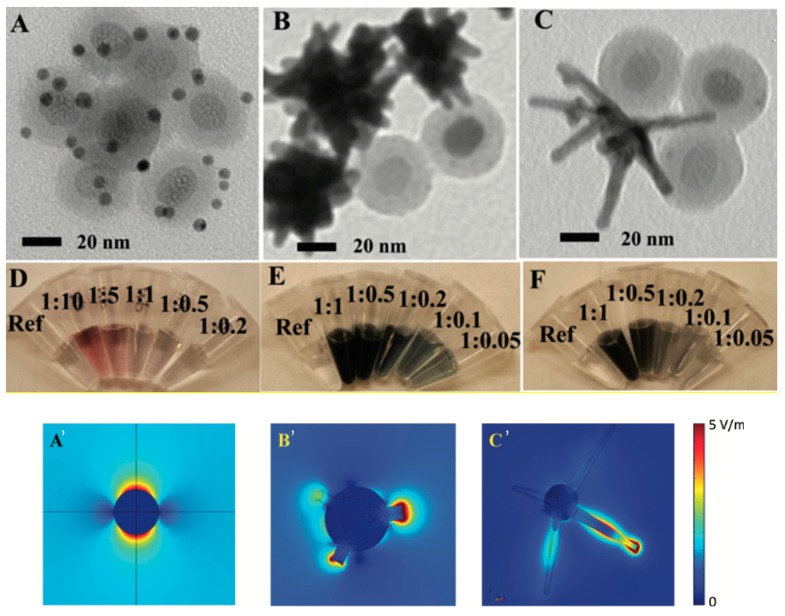
TEM images of the Au–UCNP clusters: (**A**) AuNP–UCNP, (**B**) 700 AuNS–UCNP, (**C**) 980 AuNS–UCNP and (**D**–**F**) the corresponding optical images; Images of electric field amplitude: (**A’**) AuNPs, (**B’**) AuNSs with 700 nm plasmon resonance and (**C’**) AuNSs with 980 nm [123]. Reproduced with permission. Copyright Royal Society of Chemistry, 2015.

Nanoparticles (NPs) are among the most distinguished and promising nanomaterials and have been used in a wide variety of applications (e.g., in drug delivery, gene delivery, antibacterial, catalysis, superhydrophobicity, etc.) [118,119,120,121,122]. Among the many methods to fabricate multifunctional NPs for specific purpose, self-assembly of NPs is identified as a crucial and efficient strategy that organizes discrete components into ordered structures by specific interactions. He [123] et al. prepared novel near-infrared (NIR) multifunctional Au–UCNPs via nucleobase interaction between CNA polymer-functionalized UCNPs and complementary DNA strands functionalized AuNPs (Figure 13). They demonstrated that the conjugation of AuNPs to UCNPs enhanced UCNP fluorescence, while AuNSs (nanostars) tended to quench fluorescence. Furthermore, the assembly of AuNSs-UCNP could induce rapid increases in temperature upon NIR excitation, which provides great potential for photothermal therapy.

### 4.5. Metal–Nucleobase Coordination

In nature, metal ions play important roles in the structure and function of nucleic acids. A wide variety of investigations into transition metal ions’ binding to nucleic acids have been utilized extensively, such as in disease diagnostics [124,125,126]. Other than applications in biology, the ability of nucleic acids to store and transmit information has been explored in creating nanostructures and molecular electronic devices [127,128,129]. Watson et al. [130] introduced 8-hydroxyquinoline (Q) into PNA oligomers and investigated the properties of duplexes formed by these oligomers in the presence of Cu^2+^. Their results demonstrated that the Cu^2+^-containing PNA duplexes have higher thermal stability than the unmodified PNA, and furthermore, with the help of metal ions, PNA duplex pairs exhibited high tolerance to mismatches.

**Figure 14:**
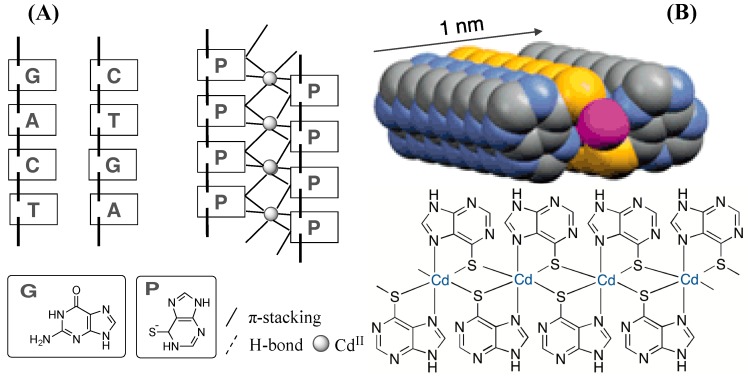
(**A**) Schematic representation of DNA and [Cd(6-MCP)_2_]_n_. (**B**) Scheme of the structure and spacefill model of [Cd(6-MCP)_2_]*_n_* [133]. Reproduced with permission. Copyright John Wiley and Sons, 2005.

Recently, coordination polymers and metal organic frameworks (MOFs) have attracted a lot of attention from researchers [131,132]. Olea et al. [133] presented a simple method to obtain single polymeric chains from crystals of [Cd(6-MCP)_2_·2H_2_O]*_n_* (6-MCP = 6-mercapto-purinate), and this particular 1D MOF exhibited significant similarities to DNA-based systems (Figure 14). García-Terán et al. [134] synthesized a 3D coordination polymer using the interaction between adenine and Cu^2+^. X-ray analysis indicated in this covalent 3D network: the centered Cu^2+^ ions are coordinated by tridentate μ-N3, N7, N9 adeninate ligands. This 3D coordination polymer has relatively large, nanometer-sized tubes and exhibited overall antiferromagnetic behavior.

## 5. Conclusions and Outlook

Although many nucleobase-containing polymers have been synthesized and applied in polymer science, the number of known nucleobase-containing polymers is still tiny compared with the whole polymer chemistry community. To achieve a broader impact and implementation in polymer and materials science, there are three directions to be pursued. Firstly, there is still a huge need to create novel nucleobase-containing molecular structures including monomers, oligomers, or polymers. Secondly, the development of novel polymeric approaches for nucleobase-containing polymers remains another critical need, especially since most synthesis of nucleobase-containing polymers lacks sequence control, which results in these nucleobase-containing polymers not usually exhibiting sequence-dependent behaviors such as we observed in nature. Finally, with the development of polymer chemistry itself, novel and more materials will be designed based on the nucleobase-containing polymers, which could provide the sequence-controlled features for these “smart” materials in widespread applications.

## Figures and Tables

**Table 1:** Common methods for the preparation of NB-containing polymers.

Polymerization method	NB-Monomer	Length	References
ATRP	Styrenes and derivatives	10~100 bases	[25,26,27]
	Acrylates		
	Methacrylates		
	(Meth)acrylamides		
	Miscellaneous monomers		
RAFT	Styrenes and derivatives	10~100 bases	[28,29,30,31,32]
	Acrylates		
	Methacrylates		
	(meth)acrylamides		
NMP	Styrenes and derivatives	20~100 bases	[33,34]
	Acrylates		
Conventional radical polymerization	Common monomers	80~200 bases	[35,36,37]
Click chemistry	Azides	10~50 bases	[38,39,40]
	Alkynes		
	Thiols		
	Vinyls		
	Acrylamides		
	Dienes		
Ring opening polymerization	Cyclic monomers (e.g., cyclic olefins, acetals, lactams, lactones)	10~40 bases	[41,42,43]
Other polymerization	Thiophenes and derivatives	N.A.	[44]
